# Hydrochromic paper-based dosimeter for monitoring UV light exposure based on the photochemical formation of gold nanoparticles

**DOI:** 10.1007/s00604-025-07020-4

**Published:** 2025-02-18

**Authors:** Tatiana G. Choleva, Vasiliki I. Karagianni, Dimosthenis L. Giokas

**Affiliations:** Department of Chemistry, University of Ioannina, 44510 Ioannina, Greece

**Keywords:** UV dosimeter, Gold nanoparticles, Solar UV exposure, Paper-based devices, Colorimetric response

## Abstract

A paper-based UV dosimeter/indicator based on the solid-state photochemical reduction of gold-cationic surfactant complexes is described. Upon exposure to UV light, the photoreductive elimination of chloride ions from the CTA-AuCl_4_^−^ complex leads to the reduction of Au ions and the formation of small Au nuclei. After hydration, the Au nuclei are washed away by capillary flow and produce gold nanoparticles by coalescence and aggregation, resulting in a blue-purple coloration on the paper surface. The reaction is initiated by  UV light of variable wavelength (from UVA at 254 nm tο UVC at 365 nm) and over a wide range of UV doses (up to 3000 mJ/cm^2^ for UVB at 312 nm and UVC at 365 nm). This versatility allows for its application in monitoring light exposure in germicidal sterilization, UV phototherapy, and as a personal solar UV indicator. The user-friendly paper facilitates both the fabrication and use of the devices, which can be activated on demand by simply wetting the paper surface.

**Graphical abstract:**

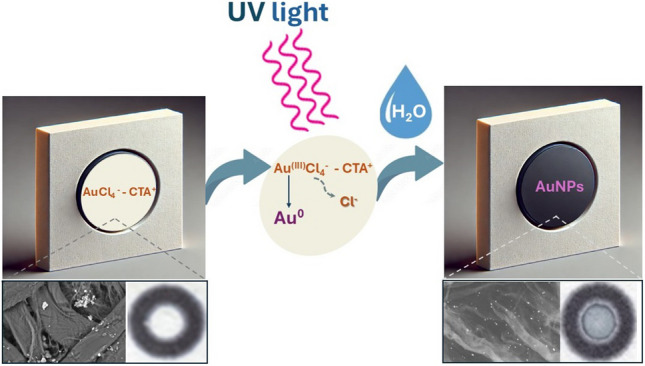

**Supplementary Information:**

The online version contains supplementary material available at 10.1007/s00604-025-07020-4.

## Introduction

Ultraviolet (UV) light is well recognized as a significant risk factor for developing skin damage and cancer [1]. Other effects of UV irradiation involve long-term damage to the cornea, retina, and lens of the eye and a weakened immune system [1–3]. Since sunlight is the primary exposure pathway to UV irradiation, significant awareness has been raised regarding the exposure to sunlight, resulting in protective (e.g., sunscreens) or preventive measures (e.g., sensors) to mitigate or monitor over-exposure and avoid UV damage ramifications.

Preventive actions against the effects of UV irradiation mainly rely on developing sensors that can measure exposure to UV irradiation and provide early warning of overexposure. Various sensory methods have been developed that rely on the photoelectric properties of semiconductor materials [4–6] or photochromic materials and molecules that produce a colorimetric response upon exposure to UV irradiation contained in sunlight [7–11]. Photoelectric devices provide accurate and fast results, and their response is reversible, meaning they can be used multiple times. However, they are mainly suited for measuring UV intensity rather than the (accumulative) dose, and they require spectral-calibrated photodetectors or spectroradiometers for their operation, which are not convenient for daily and routine use at the point of need [10]. By leveraging advances in fabrication techniques, current progress in optoelectrical technologies is directed towards miniaturized, battery-free, dosimeter-type platforms that serve as the basis for portable, autonomous, and low-cost devices [12, 13].

Although less automated, UV photosensitive materials offer many advantages pursued by advanced manufacturing of optoelectric devices (autonomous, wearable, battery-free, accumulative signal transduction, etc.). These materials exploit photo-responsive indicators that undergo photochemical transformations (oxidation/reduction, decomposition, polymerization, etc.) to initiate chromatic (coloration or discoloration) reactions [7–10, 14, 15] or photocatalyst-mediated dye degradation mechanisms [16, 16–18]. To enable their use at the point of interest, the probes are incorporated in polymeric films [7, 14], paper [10, 15, 16], or glass [8] substrates. The response of these materials is generated immediately upon exposure to sunlight, provides the cumulative dose of UV irradiation, and is usually irreversible. Notably, most of these devices are designed to respond to UV irradiation doses corresponding to the minimum erythema UVB dose for various skin types (20–200 mJ/cm^2^) but they are not suited for testing exposure to high doses of UV irradiation since they reach saturation.

Τhis work introduces a new type of UV light dosimeter probe based on the solid-state photochemical reduction of gold ions to AuNPs. The probe, incorporated in chromatography paper, consists of an aqueous mixture of AuCl_4_^−^ ions and cetyltrimethylammonium bromide as a photochemical sensitizer that is activated by UV light and photochemically reduces gold ions to AuNPs. Hydration of the paper surface produces a hydrochronic response related to the dose of UV irradiation. The colorimetric changes on the paper surface can be related to the UV dose by capturing colored images of the devices and measuring the mean grey intensity on the sensing area, or by reading by the naked eye and comparing them to pre-calibrated (reference) colored bands. The probe is responsive to cumulative doses of high-energy ultraviolet radiation, such as those used for germicidal sterilization and phototherapy of various dermatoses, as well as to sunlight.

## Experimental

### Reagents

Hydrogen tetrachloroaurate trihydrate (≥ 99.9% trace metals basis), cetyltrimethylammonium bromide, sodium citrate, sodium hydrogen, and dihydrogen phosphate were purchased from Sigma-Aldrich (Steinheim, Germany). Whatman No. 1 Chromatography sheets (20 × 20 cm, 0.18 mm, 87 g m^−2^) were obtained from Whatman (Maidstone, Kent, UK).

### Apparatus

The devices were exposed to UV light in a UV illumination chamber (Vilber Lourmat Bio-Link® BLX Crosslinker). The device can house up to five UV tubes of 8 W each (254 nm, 312, and 365 nm) and irradiate samples at a maximum distance of 15 cm. The chamber ensures constant exposure of the sensing areas of the devices to UV light throughout the experiments. It also enables control over the irradiation dose (in J/cm^2^) as a function of the UV light wavelength. Colored images of the devices were obtained with a PerfectionV370 Photo (Epson) flatbed scanner operated in reflectance mode. The images were saved in JPEG format at a resolution of 300 dpi. Natural solar light radiation data were recorded by the NOANN network of the National Observatory of Athens with a Davis Solar Radiation Sensor (6450), a precision pyranometer that detects radiation at wavelengths of 300–1100 nm [19].

### Characterization of the paper devices

Field emission-scanning electron microscopy (FE-SEM) and energy dispersive spectroscopy (EDS) studies were conducted using a Phenom Pharos G2 Desktop FEG-SEM (Thermo Fisher Scientific) equipped with an EDS detector, on samples sputter-coated with Cr. The coating was applied using a Q150T ES Plus automatic sputter coater (Quorum Technologies Ltd.), with a 5-nm layer of Cr to reduce charging before analysis. The UV–Vis diffuse reflectance spectra of the paper devices were recorded using a Shimadzu 2600i spectrophotometer, paired with an ISR-2600 Plus integrating sphere, in the wavelength range of 190–1400 nm. The reflectance data were converted to absorbance data using the Kubelka–Munk equation [20]. The ATR-IR spectra were recorded in a Perkin Elmer Spectrum Two IR. The powder X-ray diffraction patterns were recorded on a Bruker D2 Phaser X-ray diffractometer (CuKα radiation, wavelength = 1.54184 Å).

### Experimental procedure

Circular paper devices were designed in MS PowerPoint on a white background and printed in a solid-ink printer (ColorQube 8580DN, Xerox) using Whatman No. 1 chromatography paper. To melt the ink and create a hydrophilic sensing area enclosed within the hydrophobic ink circle, we heated the devices in a furnace at 140 ± 5 °C for 120 s. The devices had a diameter of 0.80 cm, of which 0.40-cm diameter was the sensing area.

**Fig. 1:**
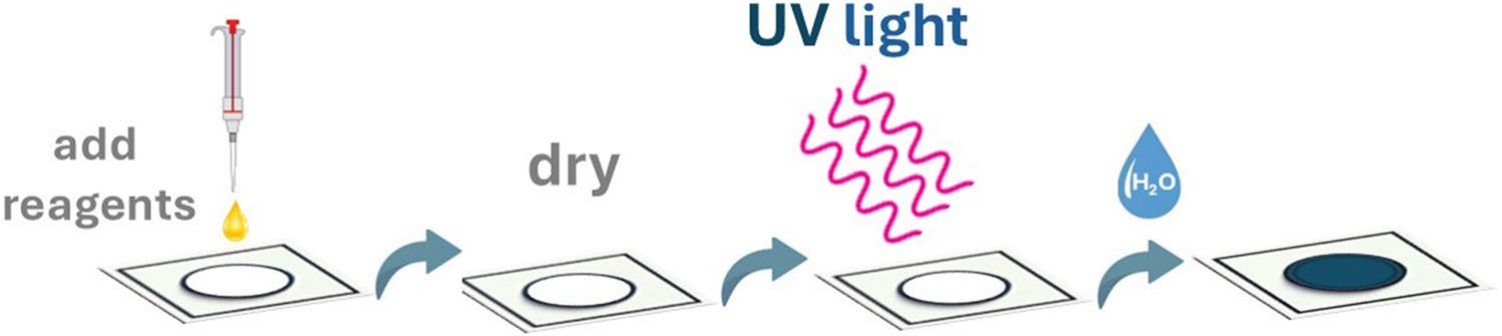
Graphical sketch of the experimental procedure for measuring UV light exposure

A mixture containing the UV-responsive probe consisting of 5.0 mM AuCl_4_^−^, 0.05 mM sodium citrate, 0.1 mM CTAB, and phosphate buffer (pH 8) was prepared before use, and 1 μL was drop cast on the center of the sensing zone using an automatic pipette. The reagents were air-dried in the dark, and the devices were irradiated with UV light. After exposure, 1 μL of distilled water was added to initiate the hydrochromic response. The devices were dried again under ambient conditions. The mean grey area intensity of the color was determined from JPEG images using Image J (NIH, USA) in the RGB color system using the embedded “oval” tool to select 90% of the total sensing area. A simplified representation of the experimental procedure is depicted in Fig. 1.

## Results and discussion

### Sensing mechanism and characterizations

The photoreduction of AuCl_4_^−^ in solution has been adequately investigated and is attributed to the excitation of Au^3+^ by UV radiation and its reduction to Au^2+^, which is unstable and quickly disproportionates to form Au^+^ and Au^3+^ [21, 22]. Then, Au^+^ may absorb another photon and be photoreduced to Au^0^ or slowly dispropriate to Au^0^ and Au^2+^. The gold atoms, Au^0^, can form gold nuclei and AuNPs, catalyzing the disproportionation reactions [21, 22]. The addition of additives (e.g., surfactants, polymers, citrate) accelerates these reactions through different chemical routes that involve either the photoreduction of the sensitizer to release electrons that reduce Au^m+^ (*m* = 1,2,3) to Au^0^ or through electron transfer reactions in the sensitizer-Au complexes [21, 23].

The photoreduction of AuCl_4_^−^ ions in a solid state and in the absence of water has not been reported. Therefore, we investigated the influence of each reagent in forming AuNPs using SEM images of the paper devices before and after exposure to UV irradiation and after wetting, which is necessary to generate the hydrochromic response. All experiments were performed at pH 8.0 using phosphate buffer. Under these conditions, Au species are distributed among various hydroxyl-containing gold complexes in the general form of (AuCl_*x*_(OH)_*y*_)^−^ (where *x* + *y* = 4) [24].

**Fig. 2:**
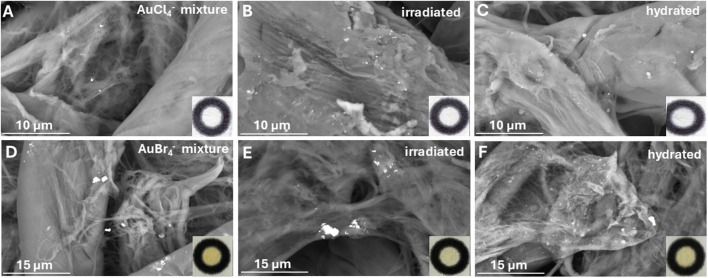
SEM images and inset photographs (bottom right corner) of the devices after the addition of reagents (**A**, **D**), irradiation at 750 mJ/cm^2^ (312 nm) (**B**, **E**), and irradiation at 750 mJ/cm^2^ (*λ* = 312 nm) and addition of 1 μL of distilled water (**C**, **F**). Devices in images **A**, **B**, and **C** contain 5 mM AuCl_4_^−^ and 0.05 mM of citrate, while devices in images **D**, **E**, and **F** contain 5 mM AuCl_4_^−^, 0.05 mM of citrate, and 200 mM NaBr

SEM images of the dry devices spiked with AuCl_4_^−^ in the presence of citrate ions but not CTAB show the appearance of sparse AuCl_4_^−^ salts, both before and after exposure to UV light, but no formation of AuNPs is observed (Fig. 2A–C). This indicates that the photoreduction of AuCl_4_^−^ does not occur in the solid state under dry conditions, which justifies the lack of a hydrochromic response on the paper surface. These observations agree with the findings of previous studies on the photoreduction of metal-citrate complexes in the absence of water [25]. Next, we investigated the photoreduction of Au-bromide salts (in the presence of citrate ions) as a potential source of AuNPs. That was decided because when CTAB is added to the AuCl_4_^−^ citrate mixture, two phenomena occur: a fast, multi-step, ligand substitution reaction of Cl^−^ from Br^−^ for anions released from the dissociation of CTAB and the formation of an ion pair between CTA^+^ and Au anions [24, 26]. To ensure that all AuCl_4_^−^ anions have been transformed to Au-Br complexes, we used a 40-fold excess of NaBr (compared to AuCl_4_^−^) so that the Au species are present in bromide/hydroxyl complexes in the general form of (AuBr_*x*_(OH)_*y*_)^−^ (*x* + *y* = 4). The presence of AuBr_4_^−^ salts on the paper devices after deposition of reagents and exposure to UV irradiation was more evident than AuCl_4_^−^ salts, possibly because they are less water soluble (Fig. 2D, E). When the devices were hydrated, a few AuNPs appeared on the paper surface, indicating that Au is photoreduced by UV irradiation when it is present as its bromide salts and then washed away by water after wetting the paper surface (Fig. 2F). However, the lack of coloration on the paper surface (Fig. 2F—inset photo) shows that the contribution of AuBr_4_^−^ in the hydrochromic response is trivial.

**Fig. 3:**
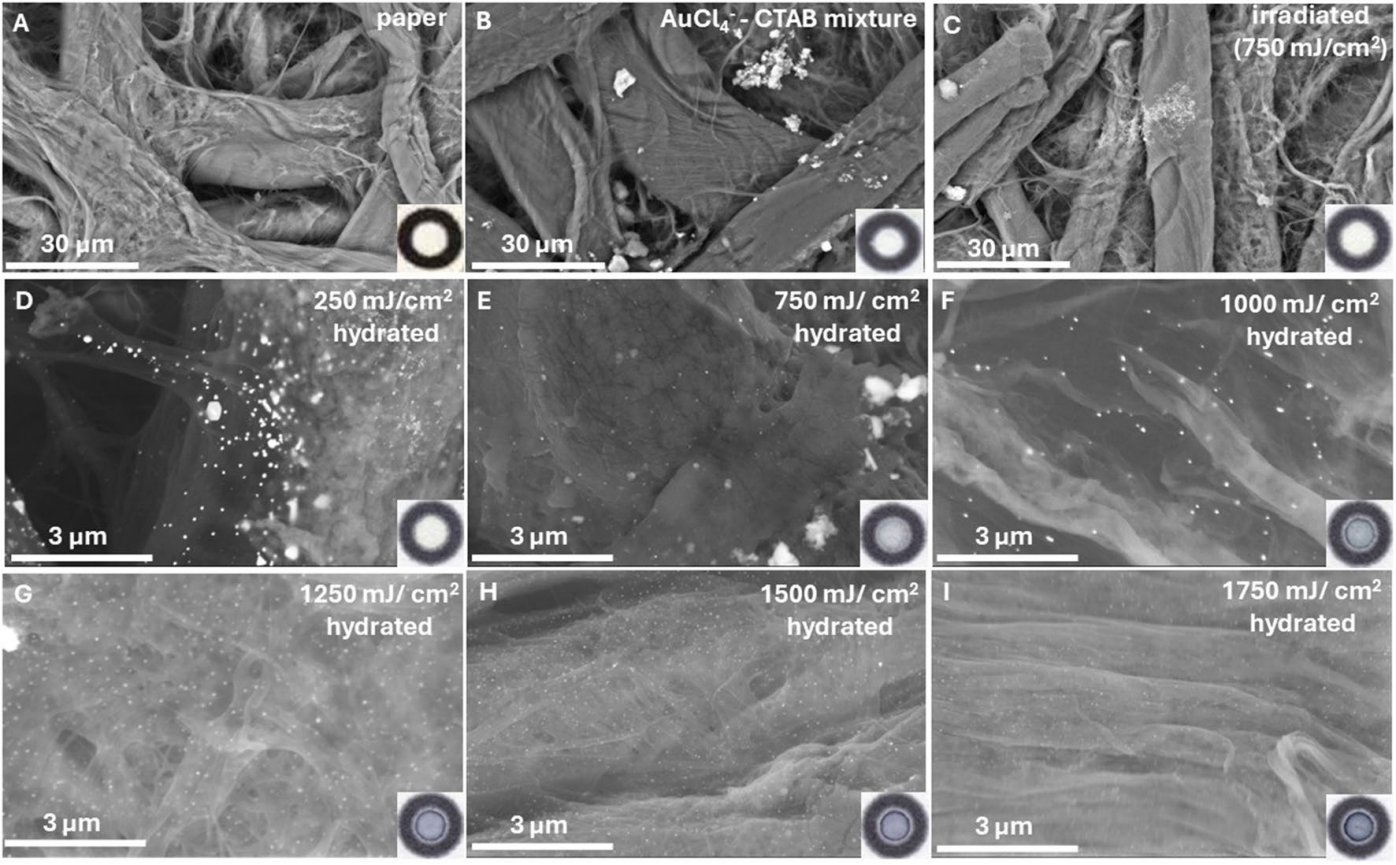
SEM images and inset photographs (bottom right corner) of the sensing zone on the **A** paper devices, **B** paper devices after the addition of the sensing probe (5 mM AuCl_4_^−^, 0.05 mM of citrate, 0.1 mM CTAB, and phosphate buffer pH 8), **C** paper devices containing the sensing probe and irradiation at 750 mJ/cm^2^ (312 nm), and **D**–**I** hydrated paper devices (1 μL of water) containing the sensing probe after irradiation with increasing radiation dose (250–1750 mJ/cm^2^, *λ* = 312 nm). Irradiation was performed in increments of 250 mJ/cm.^2^

When the AuCl_4_^−^-citrate-CTAB mixture was used as a sensory probe, an intense blue-purple coloration appeared on the paper surface after exposure to UV light and hydration of the devices. SEM images show the presence of aggregates (Fig. 3B), attributed to AuCl_4_^−^ and insoluble Au-CTAB and AuBr_4_^−^—salts (Figure S1). After irradiation, the aggregates are still visible (Fig. 3C), meaning they do not break down or dissociate by UV light. Moreover, no AuNPs can be detected. When the irradiated devices are hydrated by drop-casting distilled water, AuNPs appear, and their distribution on the paper surface increases with the irradiation dose. In parallel, the abundance of aggregates is gradually reduced (Fig. 3D–I). As aggregates disappear, more AuNPs form and disperse on the paper surface relatively homogeneously. From this observation, we infer that Au photoreduction occurs mainly on Au-CTA aggregates. Since no AuNPs are produced on the dry devices, we assume Au ions are reduced to form small nuclei, which, upon hydration, are dissolved in water, leading to their collision. This causes their aggregation into larger nanoparticles that are washed away by the action of capillary flow. This is consistent with previous findings, which reported that the growth of gold nanoparticles can be driven by coalescence and aggregation, where smaller particles contribute to the development of larger nanoparticles [27, 28]. The appearance of a colored ring on the paper surface, caused by the capillary flow that drags nanoparticles towards the droplet’s contact line (i.e., coffee ring effect) [26], provides evidence of the water-mediated formation and dispersion of AuNPs and indicates that AuNPs are not chemically bound on the paper substrate. As irradiation increases, the aggregates are photoreduced to a greater extent, and more Au nuclei are produced, forming more AuNPs. As more AuNPs become available, the overall coloration of the surface gradually becomes darker with increasing irradiation dose.

**Fig. 4:**
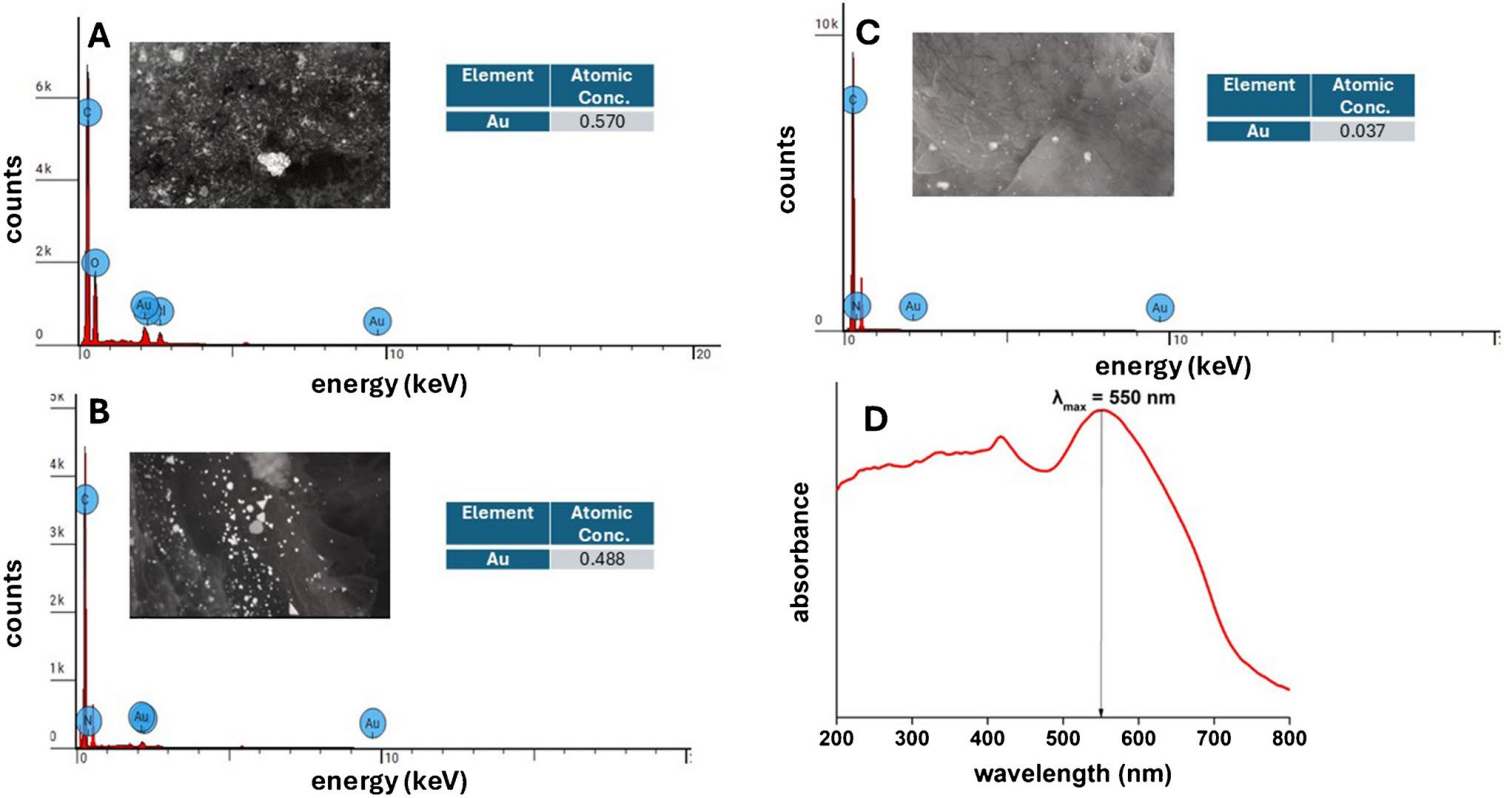
EDS spectra obtained from the paper devices containing the sensing probe (5 mM AuCl_4_^−^, 0.05 mM citrate, 0.1 mM CTAB, phosphate buffer pH 8), before (**A**), and after exposure to irradiation dose of **B** 250 mJ/cm^2^ and **C** 750 mJ/cm^2^ (*λ* = 312 nm) and hydration with distilled water. Analysis was performed in the inset images that were obtained at the same scale (2 μm) and have the same surface area of 35 μm^2^. **D** Characteristic UV–Vis (Kubelka–Munk transformed) diffuse-reflectance spectra obtained from the paper devices after exposure to irradiation and hydration

The above observations are qualitatively verified also by EDS analysis. As shown in some characteristic EDS spectra presented in Fig. 4A–C, the abundance of gold (i.e., % atomic concentration) determined in a constant surface area of the paper (35 μm^2^) decreases with an increasing irradiation dose and reaches values below 0.1% at 750 mJ/cm^2^. Similar atomic concentrations (< 0.1%) were measured also at higher irradiation doses (not shown). This is attributed to the gradual dissolution of gold-CTAB aggregates from spots with abundance of gold salts (which yield an apparent high atomic concentration of gold) and the formation and dispersion of AuNPs on a larger paper surface (yielding an apparently lower atomic concentration of gold). Moreover, the UV–Vis diffuse reflectance spectra of the irradiated and hydrated samples verify that gold is present as AuNPs as evidenced by the absorbance peak at 550 nm, which is characteristic of AuNP formation (Fig. 4D).

To elucidate if there are any differences in the crystal structure and the surface chemistry (surface-bound molecules, functional groups, etc.) of the AuNPs formed at different irradiation doses, the devices were also examined by powder XRD and ATR-IR. However, due to the low mass of reagents deposited on the surface of the paper (1 μL of the gold solution mixture corresponding to 7.5 nM AuCl_4_^−^, 0.05 nM citrate, and 0.1 nM CTAB), the powder XRD and the ATR-IR spectra were identical (Figure S2) and no additional information could be derived.

Then, we examined if residual water remaining on the paper surface during air drying of the devices under ambient conditions, contributed to reducing the Au-CTA complex. This was considered because the evaporation rate of water from surfaces is known to decrease in the presence of cationic surfactants due to the tight binding of surface water molecules with the head groups of monomer surfactant molecules through electrostatic forces [29, 30]. For this reason, we prepared a series of devices, thoroughly dried the reagents in a stream of hot air provided by a hair dryer, and immediately exposed them to UV irradiation to avoid absorbing moisture. An intense hydrochromic response was also observed, although the analytical signal was 10 ± 4% (*n* = 3) lower, indicating that traces of water may have a beneficial effect but are not responsible for forming AuNPs. Finally, the influence of citrate ions on the signal response was evaluated by preparing the devices without citrate ions. The signal was approximately 6% lower than that obtained in the presence of citrate. This value lies within the standard deviation of measurements; therefore, we intuitively considered that citrate may have a small positive effect as a photosensitizer and supports the notion that CTAB is the main photosensitizer under these conditions.

Considering that metal-citrate complexes do not undergo solid-state photoreduction under dry conditions, as discussed above, and that trimethylammonium surfactants are not photoreactive so that they can photo-oxidize and contribute to the reduction of AuCl_4_^−^ [26], we assume that the photoreduction of CTA-AuCl_4_^−^ complexes may be related to the photoreductive elimination of chloride ions from the CTA-AuCl_4_^−^ complex [31–34] leading to the reduction of Au ions and the formation of small Au nuclei. Such photoelimination reactions are feasible in the solid state [32, 34]. As water is added, the nuclei are washed away, forming larger AuNPs through coalescence and aggregation [27, 28].

### Optimization

The composition of the photosensitive reaction mixture was optimized univariately by changing one parameter at a time (concentration of AuCl_4_^−^, citrate, CTAB, and pH) while keeping the rest constant. Optimization studies were performed by exposing the devices to a radiation dose of 250–1250 mJ/cm^2^ at increments of 250 mJ/cm^2^ (corresponding to 1.7–8.6 mW/cm^2^ at increments of 1.7 mW/cm^2^) at 365 nm.

**Fig. 5:**
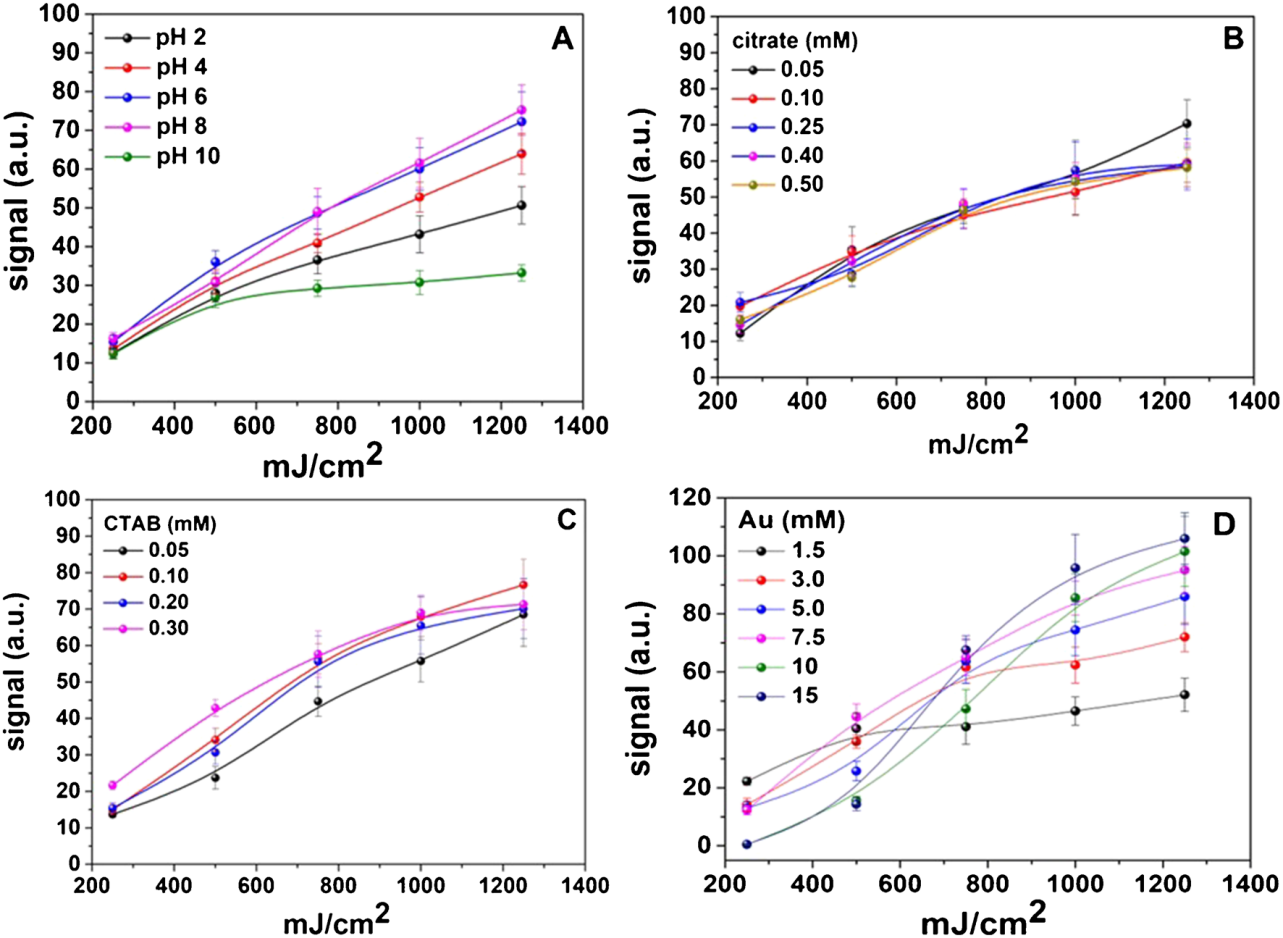
Optimization of the UV sensitivity of the probe as a function of **A** pH (experimental conditions: Au 3.0 mM, CTAB 0.05 mM, citrate 0.25 mM), **B** citrate ions (experimental conditions: Au 3.0 mM, CTAB 0.05 mM, pH = 8), **C** CTAB concentration (experimental conditions: Au 3.0 mM, citrate 0.05 mM, pH = 8), and **D** gold concentration (experimental conditions: citrate 0.05 mM, CTAB 0.1 mM, pH = 8). Irradiation was performed in increments of 250 mJ/cm.^2^

First, the influence of the pH of the aqueous gold mixture was investigated over the range of 2–10 (Fig. 5A) using dilute HCl or NaOH solutions to adjust the pH. The best photoreduction efficiency was obtained at almost neutral pH values (from 6 to 8), while acidic or too alkaline pH negatively influenced the photoreduction of Au ions to AuNPs. Acidic conditions are generally less favorable for the (photo)reduction of AuCl_4_^−^ due to several reasons related to the destabilization of AuCl_4_^−^ dissociation intermediates [35, 36] or because at acidic conditions, photolysis products (such as Cl^•^) can back-oxidize Au(0) [36]. At high pH values, the lower reduction efficiency may be attributed to the formation of [AuCl(OH)_3_]^−^ and [Au(OH)_4_]^−^, which exhibit decreased reduction potential [37, 38]. Neutral pH values favor the ligand-to-metal charge transfer (LMCT) reactions in the [AuCl_*x*_(OH)_4−*x*_]^−^ (*x* ≥ 2) complexes, which are the predominant Au species under this pH [23, 36, 38, 39]. Similar mechanisms are responsible for the photoreduction of other metal cations, such as Fe^3+^ with citric acid under UV irradiation [25, 36]. On this basis, pH 8.0 was employed using phosphate buffer (NaH_2_PO_4_/NaHPO_4_, 2.5 mM).

The addition of citrate as a photosensitizer of AuCl_4_^−^ photoreduction was investigated by varying the concentration within the range of 0.05–0.5 mM. According to the results of Fig. 5B, the maximum signal intensity of citrate ions is recorded at 0.05 mM. At higher concentrations, the signal either decreases (at lower irradiation dose) or reaches a plateau (at higher irradiation dose). The reduction of photo-efficiency with increasing carboxylic acid concentration has also been observed in other metal-citrate complexes and is attributed to the competition of carboxylate for UV light [25, 40]. Since the oxidation of citrate is a two-electron process, higher UV intensity or lower citrate concentrations are preferable to accomplish its oxidation and subsequent reduction of metal ions [25]. Therefore, 0.05 mM of sodium citrate was selected as the optimum.

In the (photo)chemical synthesis of AuNPs, cationic surfactants have been used as stabilizers and size-controlling agents [26, 41–43]. As shown in the SEM images of Figs. 3 and 4, CTAB is necessary to initiate the colorimetric response of the probe. According to the results of Fig. 5C, as CTAB concentration increases, the signal also increases due to the production of more AuNPs. Low CTAB concentrations positively influenced the formation of AuNPs, while at CTAB concentrations higher than 0.2 mM, the signal reaches a plateau with increasing irradiation dose. This phenomenon may be related to the higher sensitivity of the probe in the presence of CTAB. To avoid fast saturation of the devices and accomplish a broader response to UV light, the concentration of CTAB was set at 0.1 mM.

The concentration of Au ions was finally evaluated as the most critical factor in forming AuNPs and, consequently, in the analytical signal response (i.e., the formation of color on the paper surface). The results of Fig. 5D show that the signal increases with AuCl_4_^−^ concentration and irradiation dose while at lower AuCl_4_^−^ concentrations, a plateau is reached at a lower irradiation dose. Moreover, at AuCl_4_^−^ concentrations higher than 7.5 mM, the signal responds sigmoidally to irradiation dose which is more difficult to simulate during calibration and use. Therefore, 7.5 mM of AuCl_4_^−^ was selected as optimum at a small expense of signal intensity.

From the curves of Fig. 5, we also determined the photoreduction rate of AuCl_4_^−^ ions as a function of the components (i.e., citrate ions, CTAB, and pH) stored on paper by calculating the slope of the linear part of the curves. Student’s test (*t*-test) analysis showed that all slopes were not statistically significant, and the mean value of the slope (for each parameter separately) was at a 95% significance level, indicating that there was no significant difference in the photoreduction kinetics by varying the concentration of reagents. Similar observations have also been made in solid-state photocatalytic degradation studies which can be explained by the fact that mass transport and diffusion processes in the solid state are not rate-controlling steps as they commonly occurs in aqueous systems [17, 44].

### Selectivity

**Fig. 6:**
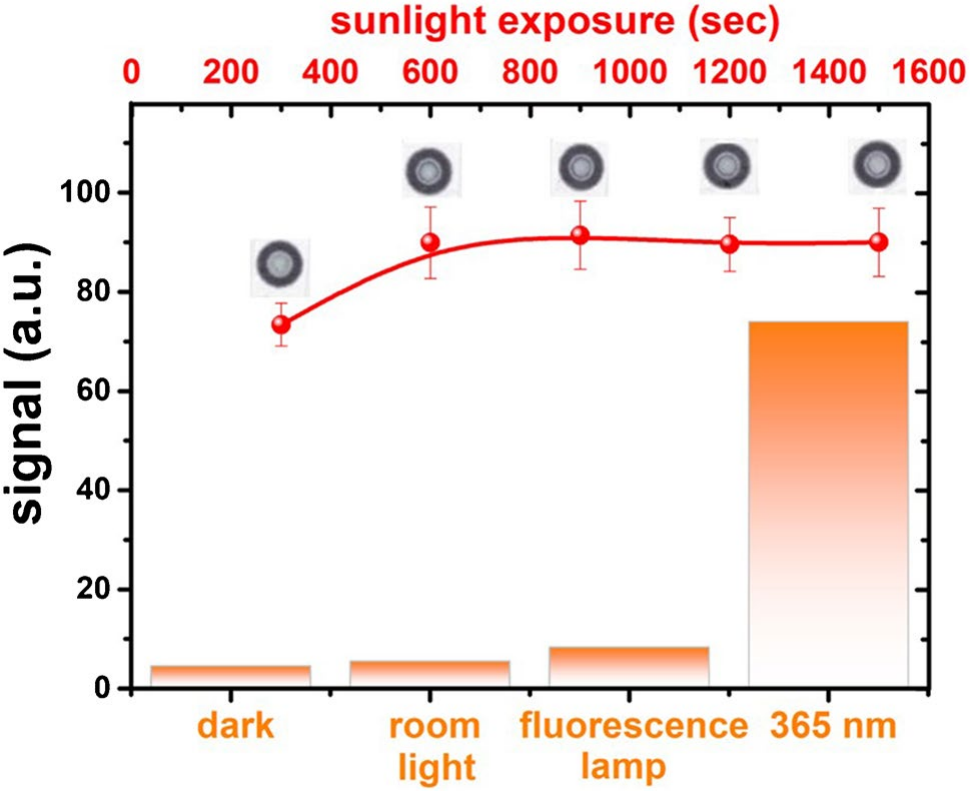
The probe’s net signal response under various light sources (orange bars) and natural sunlight (red line and circles) during summer

The selectivity of the devices was tested under different light exposure conditions such as artificial light (fluorescence tubes, cool white color), diffuse (indirect) sunlight, in the absence of light (darkness), and natural sunlight. To compare the different light conditions, all devices were exposed for a constant period of 20 min and compared to the signal intensity obtained by simultaneously exposing the devices at 365 nm. Under darkness (Fig. 6), a slight signal increase is observed, which can be attributed to the reducing properties of the paper, which may originate from cellulose and lignin, which are the main components of the paper. By subtracting the background signal generated by the paper, it can be inferred that diffuse light does not trigger the probe’s response. In contrast, exposure to artificial light causes a slight signal increase (< 3% compared to that obtained under UV light irradiation at 365 nm).

Exposure of the devices to sunlight showed a rapid response, possibly due to the high irradiation dose of sunlight. For this reason, AuCl_4_^−^ concentration was adjusted to 5.0 mM to avoid fast saturation of the devices. Under summer sunlight at noon hours, the probe produced an intense response within the first 10 min of exposure and then reached saturation. According to the solar light intensity (~ 700 W/m^2^) measured by the pyranometer at the time of exposure, the device has been exposed to 42.000 mJ/cm^2^ of sunlight irradiation within 10 min, of which 2000 mJ/cm^2^ UV light of variable wavelengths (assigning 5% of total sunlight irradiation to UV light) and 40.000 mJ/cm^2^ of visible light (> 400 nm). The high intensity of irradiation from sunlight and the combined effect of variable wavelengths, which include photons of different energy levels, may be responsible for this response. This also includes the influence of visible light since the photoreduction of AuCl_4_^−^ ions is also feasible under visible light, albeit with potentially different efficiencies and mechanisms [23, 45]. The DRS spectra obtained under sunlight were similar to those obtained under UV light, verifying the formation of AuNPs. At the same time, SEM images show the presence of AuNPs distributed on the paper surface like that observed under artificial UV light (Figure S3).

### Stability

The stability of the devices was examined by storing them in the dark within a desiccator at − 18, 4, and room temperature (20 °C) for 10 days. Due to the reduction of Au from the paper, a gradual signal deterioration was observed due to an increase of the blank (measured in devices not exposed to UV light before hydration) at room temperature. Still, it did not exceed 8% of the maximum signal. On the other hand, at 4 °C and − 18 °C, the blank signal increased by less than 4%. These data indicate that the devices maintain their stability when stored in cold and dry conditions, while under ambient conditions, the devices should be used not long after unpacking.

### Response to UV light

**Fig. 7:**
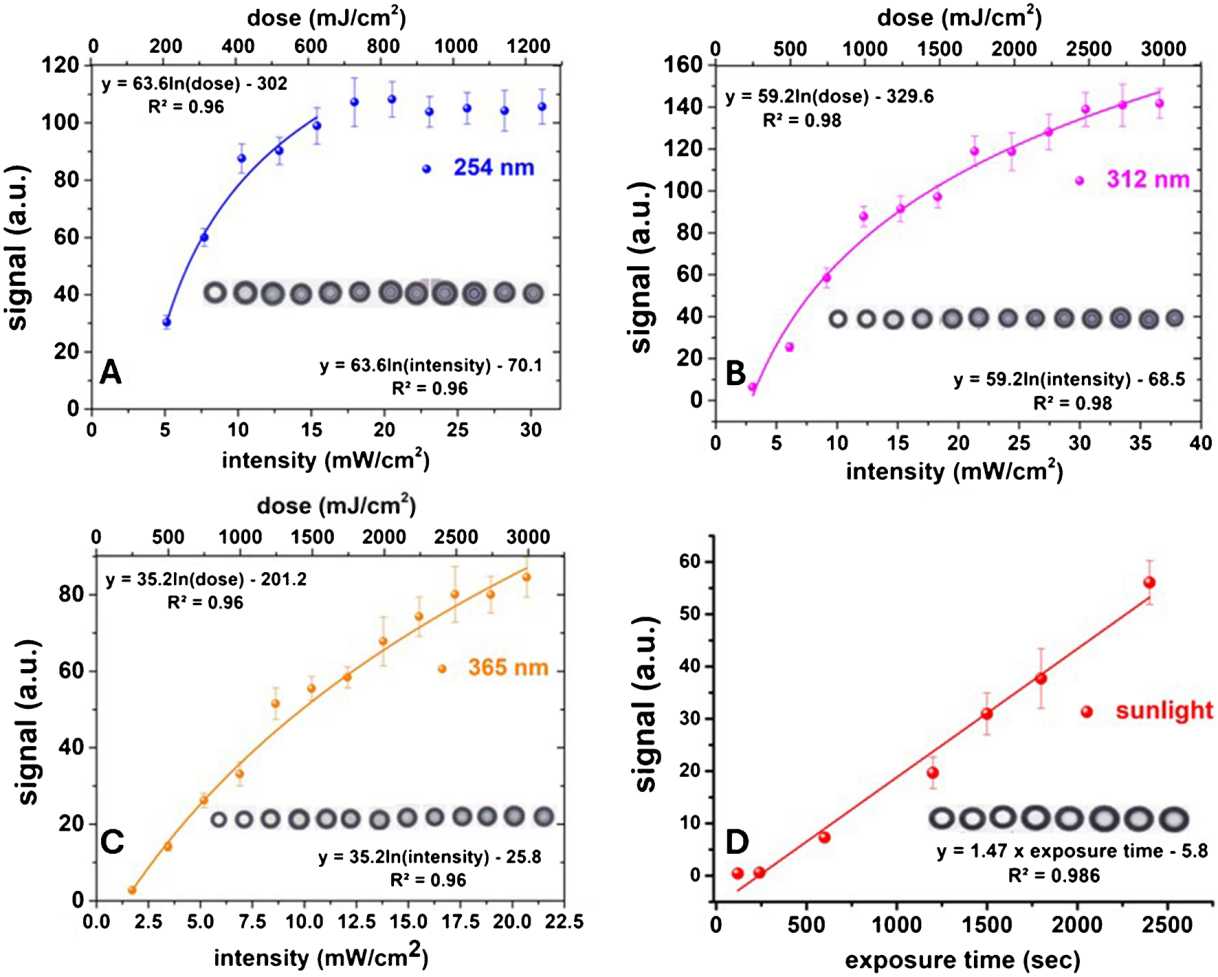
Irradiation (and intensity) dose–response curves of the paper-based UV dosimeter. **Α** 254 nm. **B** 312 nm. **C** 365 nm. **D** Natural sunlight. Inset photographs show cropped images of the devices at different exposure doses, intensities, and times

The response of the devices to various wavelengths and light irradiation was examined by exposing the devices to increasing intensity at 254-nm (UVC), 312-nm (UVB), and 365-nm (UV A) radiation wavelengths. The dose–response curves in Fig. 7 show that the devices exhibit wavelength-dependent sensitivity. The response increases logarithmically with increasing irradiation intensity (and irradiation dose), while the slope of the logarithmic equations decreases with increasing wavelength due to the lower irradiation intensity. As a result, the signal reaches saturation faster and plateaus with decreasing wavelengths. At 365 nm, the signal does not reach saturation even after receiving a 3000 mJ/cm^2^ dose, but no further doses were examined.

The developed device may be useful for various applications in controlled environments involving large doses of UV irradiation, such as medical or disinfection applications. The response of the device at 254 nm, for example, is suitable for measuring the UVC radiant energy lethal to many recalcitrant hazardous microorganisms such as bacteria, bacterial spores, fungi, viruses, and protists, some of which require high doses of UVC irradiation (e.g., *Candida auris* influenza virus) [46, 47]. It may also be used to monitor exposure during treatment of patients subjected to UVC phototherapy for various diseases, such as patients with chronic ulcers infected with methicillin-resistant *Staphylococcus aureus* (MRSA) [48] or patients exposed to UVB or UVA irradiation for the treatment of psoriasis and a wide range of other skin diseases (e.g., atopic dermatitis, eczema, vitiligo) [49–51]. The probe may also be utilized in vitro or in non-human animal model tests to evaluate the results of UV phototherapy treatment at a preclinical stage.

In addition to the above, the device may be suitable for developing a dosimeter for monitoring exposure to sunlight. To test this possibility, a proof-of-concept demonstration of the use of the device is shown in Fig. 7D. Exposure was performed on a partly cloudy winter day with a nominal sunlight intensity of ~ 500 W/m^2^. The device’s response was linear up to 40 min of continuous exposure, albeit the signal was lower than that observed in summer or under UV light (Fig. 6). The probe’s response shows that different lighting conditions produce a different response as a function not only of sunlight intensity but also of the relevant exposure conditions (diffused light intensity, stability of light intensity, etc.). Therefore, appropriate optimization of the composition of the probe and a more detailed study of the effect of various wavelengths will be required to correlate the measured response to the minimal erythema dose (MED) for different skin types. Bandpass filters on top of the devices could also enhance the selectivity of color change for UV light while reducing the influence of visible light. Although no such detailed study was performed, the response of the device was similar to other devices developed for measuring personal UV exposure to sunlight [7, 9, 10, 14–16, 52–55], indicating its potential for the development of a naked-eye, solar light UV dosimeter. The use of paper may also facilitate its adaptation into a wearable, soft, and stretchable UV photodetector device, which is activated on demand. Moreover, the low amount of reagents deposited on the paper surface is beneficial regarding fabrication cost. However, the hydrochromic response of the devices may be less convenient for routine and daily use as a solar light indicator by a non-trained user. Although only water needs to be added to generate the UV dose-dependent chromatic response, some familiarization may be necessary. Ideally, an approach that enables either the controlled hydration of the devices with minimum user intervention could mitigate this limitation.

## Conclusions

A paper-based hydrochromic photodetector that can measure the accumulated dosage of UV light was developed using the photochemical reduction of gold chloride-cationic surfactant complexes to gold nanoparticles. Radiation-driven reduction is performed in a solid state upon exposure to light. At the same time, the color changes are generated, on demand, upon hydration of the devices through the coalescence and aggregation of gold nuclei to gold nanoparticles and their distribution on the paper surface. The devices are sensitive to UV light but are not limited to a single spectral region producing a combined response when exposed to light of a variable wavelength spanning from UV to visible light without requirements for photodiodes or other electronic components. Incorporating the probe into user-friendly paper devices affords flexibility, low reagents consumption, cost-effectiveness, and easiness of operation since the end-user only has to add a drop of water to generate the response on demand. Notably, the devices are responsive over various UV doses, enabling their use in various practical applications, including germicidal sterilization, UV phototherapy, and potentially personal solar light exposure.

## Supplementary Information

Below is the link to the electronic supplementary material.

Supplementary file1 (DOCX 742 KB)

## Data Availability

No datasets were generated or analysed during the current study.
