# Supplementary material for: Hydrochromic paper-based dosimeter for monitoring UV light exposure based on the photochemical formation of gold nanoparticles
Source: Mikrochim Acta. 2025 Feb 18;192(3):169. doi: 10.1007/s00604-025-07020-4 (PMC11836219; doi:10.1007/s00604-025-07020-4)
Supplement: Supplementary file 1 — Supplementary file1 (DOCX 742 KB) [file 604_2025_7020_MOESM1_ESM.docx]

**ELECTRONIC SUPPORTING MATERIAL**

**Hydrochromic paper-based dosimeter for monitoring UV light exposure based on the photochemical formation of gold nanoparticles**

Tatiana Choleva, Vasiliki I. Karagianni, Dimosthenis L. Giokas^[[1]](#footnote-1)^*

Department of Chemistry, University of Ioannina, 44510 Ioannina, Greece

* Corresponding author. E-mail: [dgiokas@uoi.gr](mailto:dgiokas@uoi.gr)

**
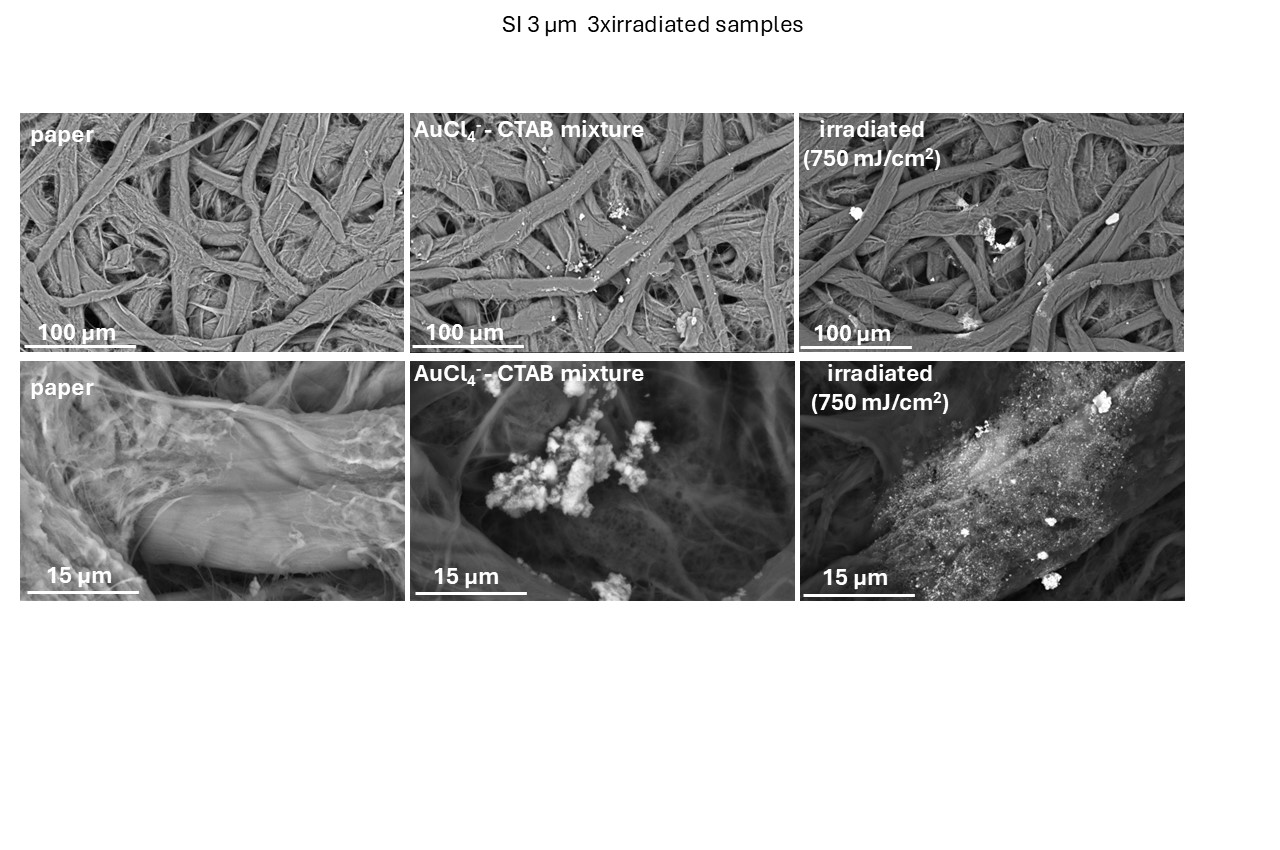
Figure S1.** SEM images of the devices shown in Figure 3A-C in lower magnification (100 μm in upper images and 15 μm in lower images).


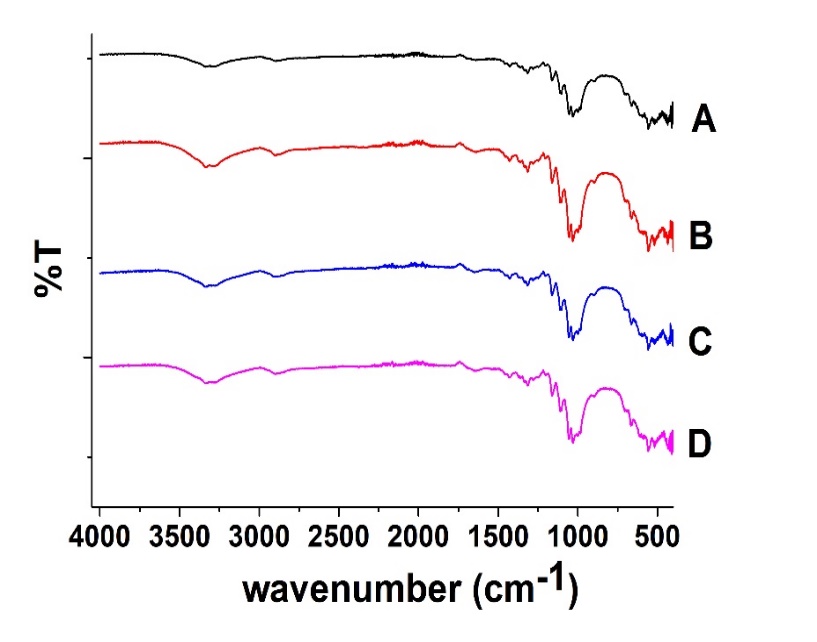

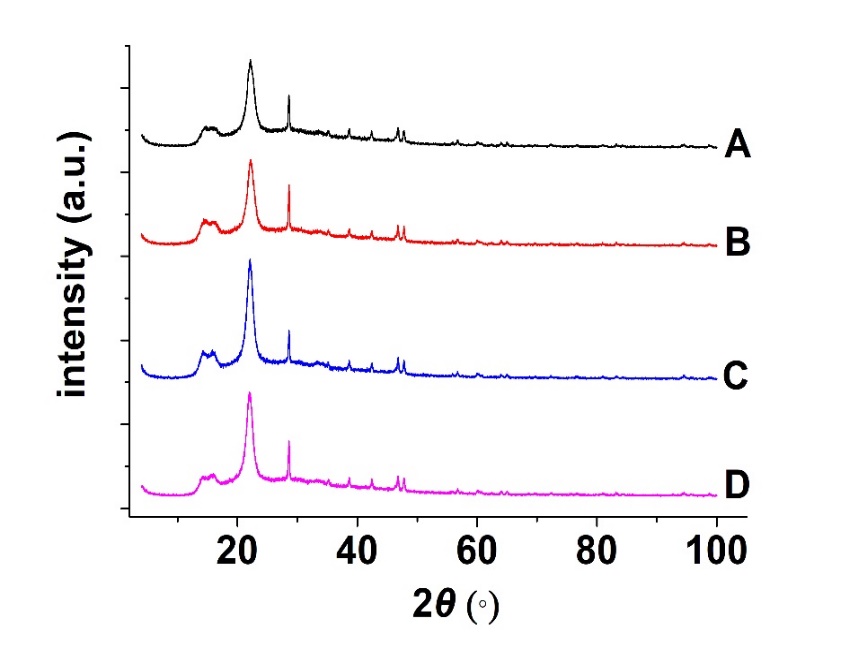


**Figure S2.** ATR-IR (left graph) and pXRD (right graph) spectra of (A) paper surface, (B) paper containing the sensing probe (5 mM AuCl_4_^-^, 0.05 mM of citrate, 0.1 mM CTAB and phosphate buffer pH 8), (C) paper containing the sensing probe after exposure to 750 mJ/cm^2^ (λ=312 nm) and (D) hydrated paper containing the sensing probe after exposure to 750 mJ/cm^2^ (λ=312 nm).


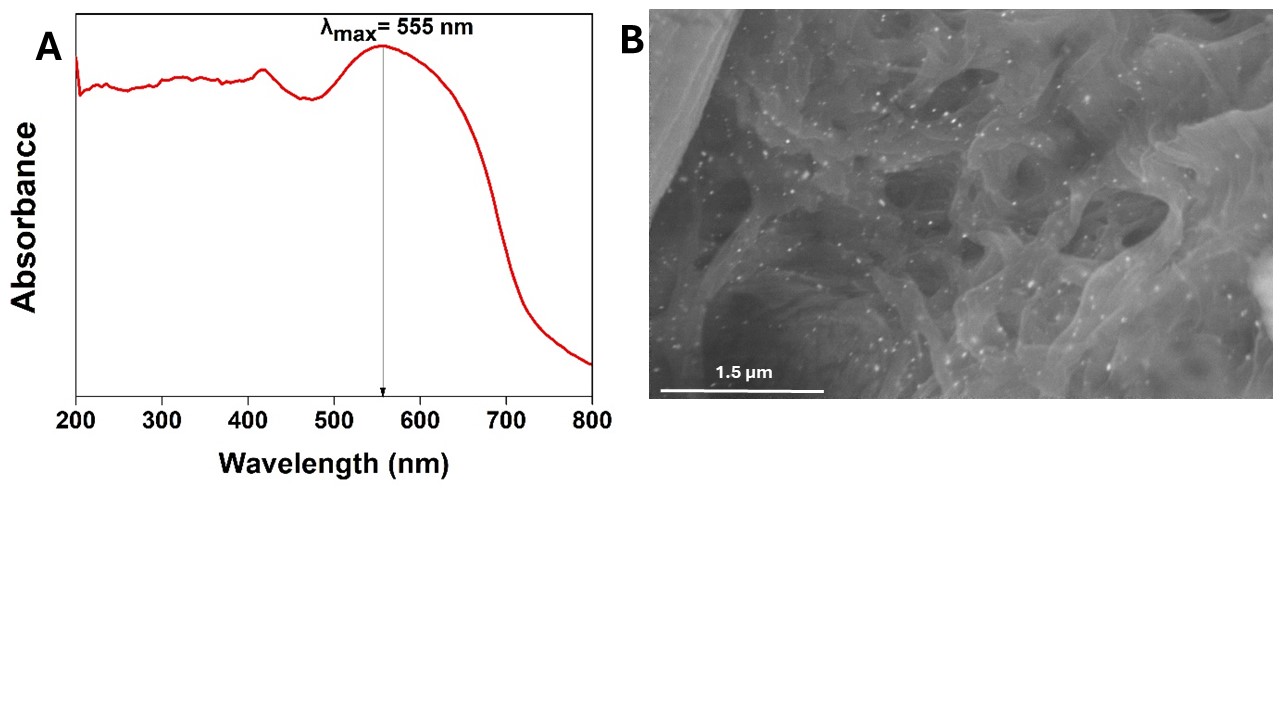


**Figure S3.** (A) UV–Vis (Kubelka–Munk transformed) diffuse-reflectance spectra and (B) SEM images, obtained from the paper devices exposed to sunlight for 30 minutes.

1. * Corresponding author. E-mail: [dgiokas@uoi.gr](mailto:dgiokas@uoi.gr) [↑](#footnote-ref-1)
